# Immunological Properties of Murine Parthenogenetic Stem Cell-Derived Cardiomyocytes and Engineered Heart Muscle

**DOI:** 10.3389/fimmu.2017.00955

**Published:** 2017-08-14

**Authors:** Michael Didié, Satish Galla, Vijayakumar Muppala, Ralf Dressel, Wolfram-Hubertus Zimmermann

**Affiliations:** ^1^Institute of Pharmacology and Toxicology, University Medical Center Göttingen, Göttingen, Germany; ^2^Clinic for Cardiology and Pneumology, University Medical Center Göttingen, Göttingen, Germany; ^3^DZHK (German Center for Cardiovascular Research), Partner Site Göttingen, Göttingen, Germany; ^4^Institute of Cellular and Molecular Immunology, University Medical Center Göttingen, Göttingen, Germany

**Keywords:** pluripotent stem cells, engineered heart muscle, tissue engineering, cardiomyocytes, parthenogenesis, cytotoxic T lymphocytes, MHC class I molecules, immunology

## Abstract

Pluripotent parthenogenetic stem cells (pSCs) can be derived by pharmacological activation of unfertilized oocytes. Homozygosity of the major histocompatibility complex (MHC) in pSCs makes them an attractive cell source for applications in allogeneic tissue repair. This was recently demonstrated for pSC-based tissue-engineered heart repair. A detailed analysis of immunological properties of pSC-derived cardiomyocytes and engineered heart muscle (EHM) thereof is, however, lacking. The aim of this study was to determine baseline and cytokine-inducible MHC class I and MHC class II as well as programmed death ligand-1 (PDL-1) and co-stimulatory protein (CD40, CD80, CD86) expression in pSC-derived cardiomyocytes and pSC-EHM *in vitro* and *in vivo*. Cardiomyocytes from an MHC-homologous (H2^d/d^) pSC-line were enriched to ~90% by making use of a recently developed cardiomyocyte-specific genetic selection protocol. MHC class I and MHC class II expression in cardiomyocytes could only be observed after stimulation with interferon gamma (IFN-γ). PDL-1 was markedly upregulated under IFN-γ. CD40, CD80, and CD86 were expressed at low levels and not upregulated by IFN-γ. EHM constructed from H2^d/d^ cardiomyocytes expressed similarly low levels of MHC class I, MHC class II, and costimulatory molecules under basal conditions. However, in EHM only MHC class I, but not MHC class II, molecules were upregulated after IFN-γ-stimulation. We next employed a cocultivation system with MHC-matched and MHC-mismatched splenocytes and T-cells to analyze the immune stimulatory properties of EHMs. Despite MHC-mismatched conditions, EHM did not induce splenocyte or T-cell proliferation *in vitro*. To evaluate the immunogenicity of pSC-derived cardiomyocytes *in vivo*, we implanted pSC-derived embryoid bodies after elimination of non-cardiomyocytes (cardiac bodies) under the kidney capsules of MHC-matched and -mismatched mice. Spontaneous beating of cardiac bodies could be observed for 28 days in the matched and for 7 days in the mismatched conditions. Teratomas formed after 28 days only in the MHC-matched conditions. Immunohistochemistry revealed single clusters of CD3-positive cells in the border zone of the implant in the mismatched conditions with few CD3-positive cells infiltrating the implant. Taken together, MHC-matched pSC-cardiomyocyte allografts show little immune cell activation, offering an explanation for the observed long-term retention of pSC-EHM allografts in the absence of immunosuppression.

## Introduction

Soon after the first derivation of human embryonic stem cells (1) and the introduction of human induced pluripotent stem cells [iPSCs; (2)], the potential of pluripotent stem cells in cell replacement therapies had been realized and enthusiastically investigated. Several clinical trials, mostly on the use of embryonic stem cells derivatives in degenerative diseases such as macular degeneration, type I diabetes mellitus, Parkinson’s disease and heart failure are underway [for an overview see Ref. (3)]. In contrast to embryonic stem cells, iPSCs may be directly derived from patients for autologous applications without the need for immune suppression. This idea was supported by studies demonstrating that terminally differentiated grafts derived from embryonic stem cells and iPSCs are not rejected in syngeneic recipients (4–6). However, in one study, iPSC autografts, in contrast to initial expectations, induced immune responses (7). These contradictory results could be explained by the finding that the immunogenicity of autologous grafts appears to depend on the cell types into which pluripotent stem cells have been differentiated (8). Because of the high risk of production failure associated with the preparation of personalized autografts, it appears, despite the potential immunological advantage, unlikely that iPSC-derived autografts will be streamlined into clinical application, especially in large patient cohorts with a medically unmet need for therapy such as in patients diagnosed with heart failure. This caveat is further underscored by the halt of the first iPSC autograft study aiming at the repair of macular degeneration despite encouraging data in a single patient (9) and its restart as an allograft trial. These data collectively indicate that a more detailed characterization of pluripotent stem cell transplant immunology is needed to harness their full therapeutic potential (10). Consensus exists however that in allograft studies, homozygosity of the major histocompatibility complex (MHC) would be advantageous (11) and global efforts are on the way to create so called haplobanks comprising HLA homozygous iPSCs for allografting (12).

Parthenogenetic stem cells (pSCs), generated from pharmocologically activated oocytes have come into focus as an alternative pluripotent cell source for cell-based therapies, because they typically exhibit HLA homozygosity (13, 14). The creation of haplobanks comprising HLA homozygous pSCs with a comprehensive coverage of putative recipient HLA types should be straight forward, based on simulations of the number of HLA haploidentical donors needed to match up the UK population (15). pSCs exhibit similar pluripotency as reported for embryonic stem cells and iPSCs and have already been tested with encouraging results in preclinical studies for the treatment of liver failure due to deficiency of fumarylacetoacetate hydrolase, beta-thalassemia, Parkinson’s disease, and skin defects (16–19). We have previously demonstrated that pSC-derived cardiomyocytes can be used for the construction of engineered heart muscle (EHM) with properties of native myocardium and shown evidence for its application in the augmentation of heart function in a mouse model of heart failure (20). Importantly, MHC-matched pSC-EHM allografts were retained without the need for comprehensive immune suppression—only methylprednisolone was applied to attenuate immune responses. The functional support observed in this study was in agreement with our earlier findings in a rat allograft study, which was performed without MHC-matching and under immune suppression with cyclosporine, azathioprine, and methylprednisolone (21).

In this study, we sought to determine baseline and cytokine-induced expression of MHC class I and class II molecules as well as several immunomodulatory molecules (PDL-1, CD40, CD80, CD86) in pSC-derived cardiomyocytes and EHM *in vitro* and *in vivo*. We submit that a more detailed understanding of the immunological properties of pSCs will be instrumental as to their translation into an off-the-shelf type allograft therapeutic candidate.

## Materials and Methods

### Neomycin Selectable pSC Lines

Parthenogenetic stem cells were generated as described before (20). In short, oocytes were harvested from superovulated 6- to 8-week-old (C57BL/6J × DBA/2J)F1 (B6D2F1) mice and activated with strontium-chloride for 6 h. Second polar body extrusion was prevented with cytochalasin B (5 µg/ml). pSCs were derived from the inner cell masses and transfected with a linearized plasmid encoding for a neomycin-resistance (NeoR) under control of a cardiomyocytes-specific 5.5 kb α-myosin-heavy-chain (αMHC) promoter fragment and a hygromycin resistance under the control of ubiquitously active phosphoglycerate kinase promoter (kindly provided by Prof. Loren J. Field, Indianapolis, IN, USA).

### Culture and Differentiation of pSCs

Parthenogenetic stem cell-colonies were cultured on inactivated murine embryonic fibroblasts with stem cell medium (DMEM, 15% FCS, 1,000 U/ml LIF, 2 mmol/L L-Glutamine, 1× non-essential amino acids (NEAA), 50 U/ml Penicillin, 50 µg/ml Streptomycin, 1 mmol/L Na^+^-Pyruvate, 1× nucleoside mix (in μmol/L: 30 adenosine, 30 guanosine, 30 cytidine, 30 uridine, 10 thymidine), and 100 µmol/L 2-mercaptoethanol) as described previously (20). pSC colonies were dissociated into single cells by trypsinization, preplated to reduce the murine embryonic fibroblast content, and resuspended with differentiation medium (Iscove Medium, 20% FCS, 2 mmol/L L-glutamine, 1% NEAA, 100 U/ml Penicillin, 100 µg/ml Streptomycin, 100 µmol/L 2-mercaptoethanol, 0.02 mmol/L L-ascorbic acid 2-phosphate sesquimagnesium salt hydrate). The cell suspension was then transferred into a 125 ml culture vessel (Techne, F7988) containing 25 ml of constantly stirred differentiation medium to generate embryoid bodies. On day 11, 200 µg/ml of geneticin (G-418) was added to the medium for the selection of cardiomyocytes. After 7 days of selection, cardiac bodies were harvested and digested into single cardiomyocytes with collagenase I and DNAse I for further experiments.

### Preparation of EHM

Custom-made glass culture dishes consisting of 4 ring-shape molds (inner/outer diameter: 4/10.6 mm) were used for casting EHMs. The volume inside each mold was 450 µl. All the pipetting steps were carried out on ice to prevent premature polymerization of the EHM reconstitution mixture. 1.5 × 10^6^ cell mixture containing 70% of pSC-derived cardiomyocytes and 30% non-cardiomyocytes (inactivated murine embryonic fibroblasts) were mixed and resuspended with differentiation medium and collagen type I (0.4 mg/EHM). 450 µl of mixture was cast into each mold and incubated at 37°C in a humidified incubator with 5% CO_2_ for 1 h. After initial condensation of the collagen, 6 ml of differentiation medium was added into each glass culture dish. The EHMs were incubated at 37°C in a humidified incubator with 5% CO_2_ for three days. On day 3, EHMs were transferred to static stretchers and kept in culture for 10 more days. Medium was changed every other day during the entire culture time.

### Isometric Contraction Measurements

On culture day 12, EHMs were suspended between retaining hooks and force transducers inside organ baths containing Tyrode’s solution at 37°C under electrical stimulation at 4 Hz with pulse duration of 5 ms and 200 mA current. pH was adjusted to 7.4 with carbogen (95% O_2_, 5% CO_2_ gas mixture). EHMs were initially pre-stretched under 1.8 mmol/L [Ca^2+^] until the force reached a stable maximal level. Contraction forces were measured under cumulatively increasing [Ca^2+^] (0.2–2.8 mmol/L). The maximum and minimum forces were measured by PC-based acquisition software (BMON, Engineering firm G. Jaeckel, Hanau). Force of contraction was calculated by the difference between maximum and minimum forces.

### Flow Cytometry of Immunological Molecules

Mouse recombinant IFN-γ (Peprotech) was added to unselected embryoid and cardiac bodies cultured in Petri dishes at a concentration of 25 ng/ml and incubated at 37°C in a humidified incubator with 5% CO_2_ for 48 h. After 48 h, these embryoid and cardiac bodies were dissociated with 6 ml collagenase and 20 µl/ml DNAse for 1 h at 37°C. The cells were passed through a 70 µm cell strainer to remove cell clumps. Cell-surface antigen expression was detected using fluorescently conjugated antigen-specific antibodies directed against MHC class I molecules (PE anti-mouse H-2Kd: clone SF1-1.1, PE anti-mouse H-2Kb: clone AF6.88.5, Biolegend), MHC class II molecules (I-A/I-E; BDbiosciences), CD1d (Biolegend), CD40 (Biolegend), CD80 (Biolegend), CD86 (Biolegend), programmed death-1 ligand (PD-L1) (Biolegend), and PD-1 (Biolegend). Labeling of the cells with unconjugated primary antibodies (α-actinin, clone EA-53, Sigma; cardiac-troponin I, clone ab47003, abcam; myosin heavy chain, clone MF-20, DSHB) was followed by incubation with fluorescently tagged secondary antibodies (anti-mouse Alexa Fluor^®^ 546, anti-rabbit Alexa Fluor^®^ 488, anti-mouse Alexa Fluor^®^ 633; Molecular Probes) at 4°C for 30 min. After staining, the cells were analyzed by flow cytometry (fluorescence activated cell sorting, FACS). Cells were gated on forward and side scatter dot plots. 10,000 events per sample were acquired and the data were analyzed with flowing software (free software version 2.5.1). Hoechst (BD biosciences) or sytox (life technologies) was used for gating of viable cells.

### Immunofluorescence Imaging

Cardiomyocytes seeded on coverslips and EHMs as whole mounts were fixed with acid free (pH 7), phosphate-buffered formaldehyde (4%) solution. The cells and EHMs were permeabilized and the unspecific binding sites were saturated by incubation with blocking buffer containing 5% FBS, 1% bovine serum albumin (BSA) and 0.5% TritonX-100 in DPBS for 30 min (cells) or overnight (EHMs) at room temperature. Cells and EHMs were incubated with primary antibodies against α-actinin (clone EA-53, Sigma), cardiac-troponin I (clone ab47003, abcam), connexin 43 (clone 610062, BD Pharmingen) for 60 min (cells) or overnight (EHMs) at room temperature. Subsequently, incubation with secondary antibody conjugated with fluorescent dye (anti-mouse Alexa Fluor^®^ 546, anti-rabbit Alexa Fluor^®^ 488, anti-mouse Alexa Fluor^®^ 633; Molecular Probe) was performed for 60 min (cells) or overnight (EHMs) at room temperature. Nuclei were counterstained with 4′,6′-diamidino-2-phenylindole or Hoechst 33342 dye. Fluorescent images were acquired using a laser scanning confocal microscope (LSM710, Zeiss, Germany).

### Isolation of Mouse Splenocytes

Mice (C57BL/6J and DBA/2J) were sacrificed by cervical dislocation and dissected under sterile conditions. Spleens were collected in a dish containing 1x PBS, crushed and rinsed through a 70 µm filter. Red blood cell lysis was done by incubating the splenocytes with 1× Red Blood cell lysis buffer (Biolegend) for 5 min at room temperature. The remaining splenocytes were then washed and resuspended in 10% RPMI and counted in a Neubauer chamber.

### Isolation of T-Lymphocytes from Spleen

T-lymphocytes were isolated from splenocytes by using the mouse Pan T cell isolation kit II (Miltenyi biotec 130-095-130). The cells were counted using a Neubauer chamber. T-cell purity was measured by flow cytometry.

### Immune Cell Proliferation Assay

Splenocytes were washed with PBS, resuspended in 5 µmol/L solution of Cell Proliferation Dye eFluor^®^ 670 (ebioscience) in PBS and incubated for 10 min at 37°C in the dark. The eFluor^®^ 670 dye labeling was stopped by adding 4–5 volumes of cold RPMI with 10% FBS and incubated on ice for 5 minutes. 1 × 10^6^ eFluor^®^ 670 labeled splenocytes were cocultivated with EHMs for 4 days on a shaker with RPMI medium at 37°C and 5% CO_2_. After 4 days of coculture, splenocytes were collected and filtered through a 70 µm cell strainer, stained with an Alexa 488-labeled anti-CD3 antibody (Biolegend) and the proliferation of CD3-positive splenocytes was analyzed using flow cytometry. The percentage of proliferating cells was calculated by the number of cells with a fluorescence intensity between labeled unstimulated cells and stimulated unlabeled control cells in relation to the cell number of unstimulated cells.

### Animal Experiments

All animal experiments were performed according to institutional and governmental guidelines and approved by the Niedersächsisches Landesamt für Verbraucherschutz und Lebensmittelsicherheit (LAVES).

#### Cell Implantation under the Kidney Capsule

Parthenogenetic stem cell-derived cardiac bodies were implanted under the kidney capsule (right kidney) of B6D2F1 (H2^b/d^) and C57BL/6J (H2^b/b^) mice (8–15 weeks age). The implantation was performed under isoflurane (3%) anesthesia. A vertical incision was made at the right flank of the animal and the kidney was pulled out gently. A small tear was made in the kidney capsule with the help of a sharp needle. Embryoid and cardiac bodies were gently placed under the capsule via a blunt 21 G cannula. The tear of the kidney capsule was then cauterized and the kidney was carefully relocated retroperitoneally. The incision was closed with a 6-0 Prolene^®^ (Ethicon) suture followed by suturing the skin. Subsequently animals were placed on a warm heating pad. For analgesia, buprenorphine (0.1 mg/kg) was administered subcutaneously. Kidneys were harvested at days 1, 3, 7, 14, 28, and 56 and immediately examined for beating areas using a microscope (Zeiss, Lumar.V12, SteREO) and proceeded further for histological analysis.

#### Histological Analysis

Explanted kidneys from the mice were fixed in 4% formaldehyde (in PBS), embedded in paraffin. 4 µm sections were used for hematoxylin eosin or CD3 immune staining. For CD3 staining, the sections were deparaffinized. Antigen retrieval was performed by boiling the slides in sodium citrate buffer (10 mmol/L sodium citrate, pH 6, 0.05% Tween 20). The sections were blocked with 4% BSA/PBS followed by incubation over night at 4°C with an anti-CD3 antibody (ABD Serotec, MCA1477). After washing, the endogenous peroxidase activity was blocked with 3% H_2_O_2_ for 10 min. A biotinylated goat anti-rat IgG secondary antibody (Dianova, 112-065-062) was added for 45 min at room temperature. After washing, horseradish peroxidase-conjugated streptavidin (Biolegend, 405210) was added and slides were incubated for 30 min at room temperature followed by three washing steps of the DAB substrate for 3–5 min. The slides were then counterstained with hematoxylin. The slides were imaged with an upright microscope (Zeiss, Imager.M2).

### Statistics

GraphPad Prism software (GraphPad Software Inc; San Diego) was used to convert data sets into graphs (displayed as mean ± SEM) and subjected to Student’s *t*-test or one-way ANOVA with a suitable *post hoc* test. A *p* < 0.05 was considered to indicate significance. Sample number (*n*) and statistical test are presented with each data set.

## Results

### Differentiation of MHC-I-Homozygous pSC into Cardiomyocytes

**Figure 1:**
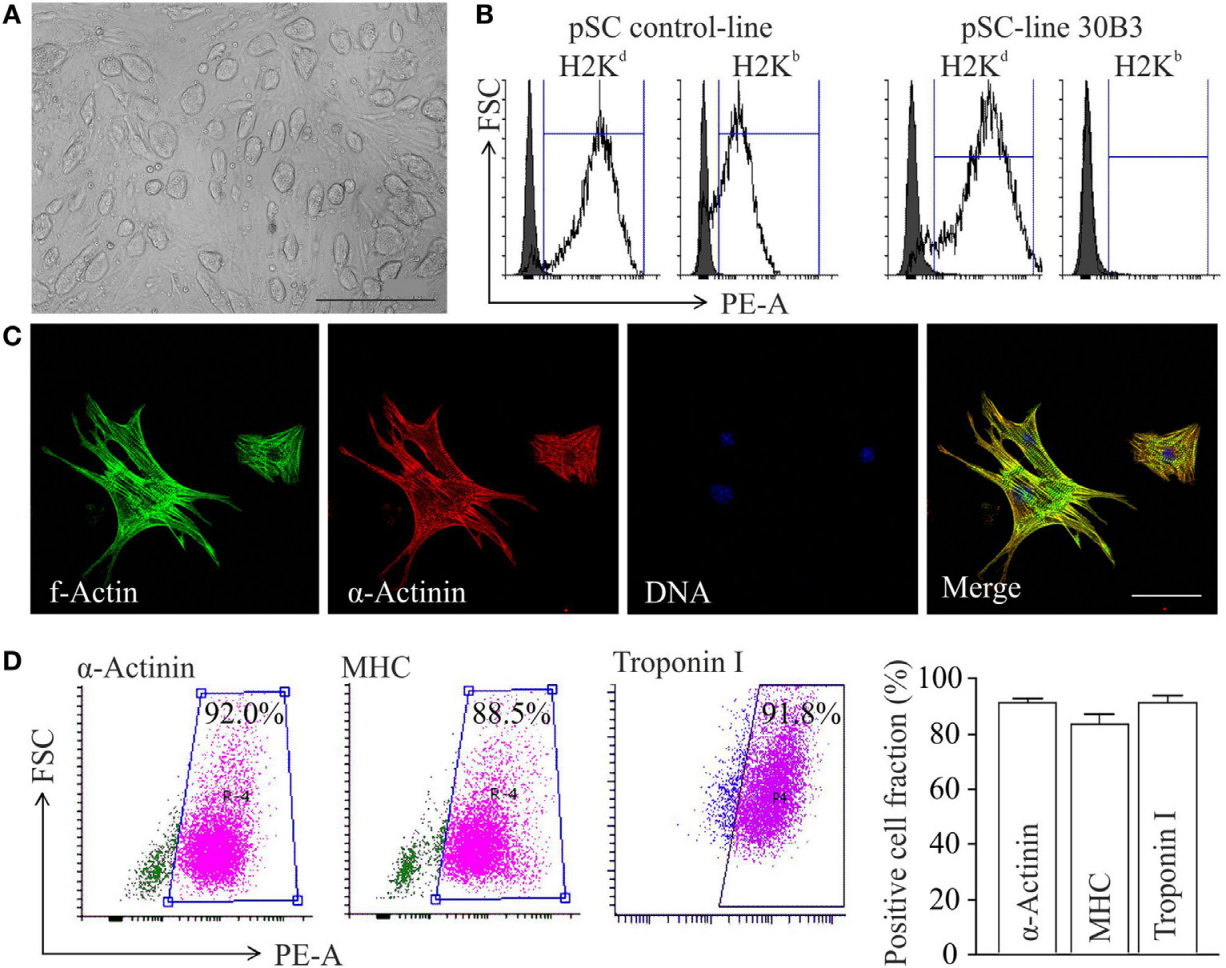
Differentiation of major histocompatibility complex (MHC)-I-homozygous parthenogenetic stem cells (pSCs) into cardiomyocytes. **(A)** Morphology of undifferentiated pSC colonies at passage 21 cultured on murine embryonic fibroblasts. Scale bar: 500 µm. **(B)** FACS analysis of MHC class I molecules on unselected derivatives of an MHC-heterologous control pSC-line (left panel) and on the MHC-homologous pSC30B3-line used in this study (right panel), both generated from oocytes derived from the MHC-heterologous B6D2F1 mouse-strain. **(C)** Confocal microscopy of pSC-derived cardiomyocytes. Scale bars: 20 µm. **(D)** FACS-analysis of cardiomyocyte-specific proteins in pSC-derived cardiomyocytes after 7 days of chemical purification with G418 (MHC: myosin heavy chain). Left panels: Representative scatter blots. Right panel: Summary of the flow cytometry data (*n* = 4–12/group).

Undifferentiated cell colonies from the pSC cell-line 30B3, carrying a NeoR under the control of the cardiomyocyte-specific α-myosin-heavy-chain promoter (αMHC-Neo) (22), showed a morphology typical for undifferentiated pluripotent stem cells (Figure 1A). This cell line was generated from an MHC heterozygous (H2^b/d^) C57BL/6J × DBA/2J F1 (B6D2F1) hybrid oocyte donor. MHC homozygosity (H2^d/d^) confirmed by FACS (Figure 1B) is typical feature of pSCs (20). After propagation and spontaneous differentiation in spinner-flasks non-cardiomyocytes were eliminated by G418-treatment. The resulting cardiomyocyte population (Figure 1C) showed a purity of ~90% (Figure 1D).

### Expression of Cell Surface Markers with Immune Functions on pSC-Derived Undirectedly Differentiated Cells and pSC-Derived Cardiomyocytes

**Figure 2:**
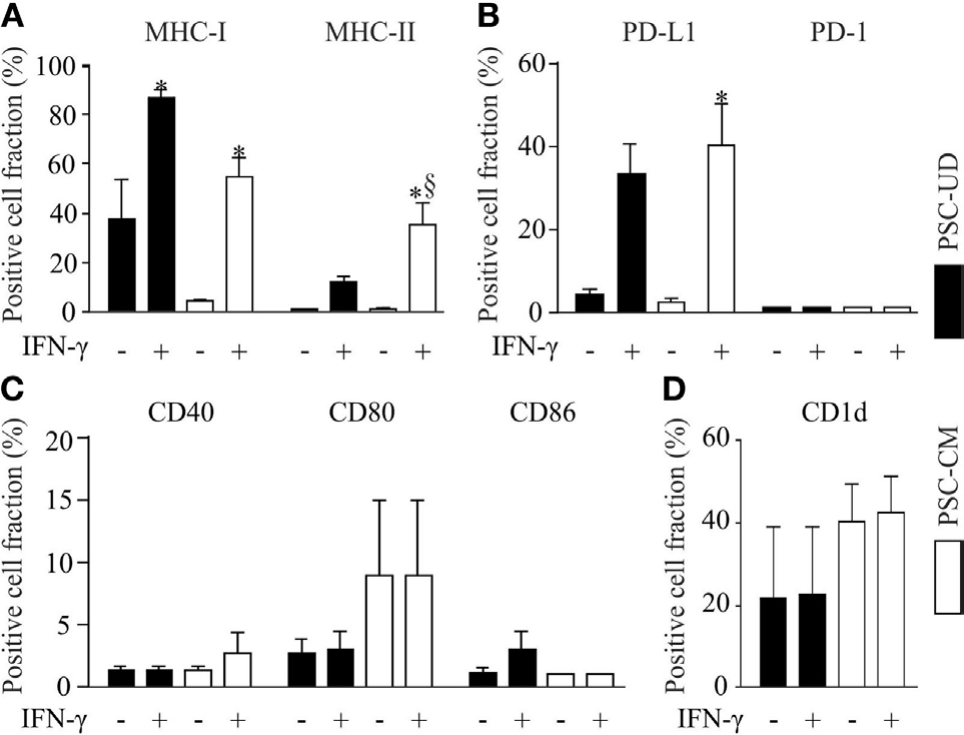
Flow cytometric analysis of cell surface markers with immune functions on undirectedly differentiated pSC-derivatives (pSC-UD) and pSC-derived cardiomyocytes (pSC-CM) with and without IFN-γ-stimulation. Cell-fractions positive for major histocompatibility complex (MHC) class I and MHC class II molecules **(A)**, for programmed death ligand-1 (PDL-1) and PD-1 **(B)**, for co-stimulatory molecules CD40, CD80, and CD86 **(C)** and for CD1d **(D)**. *p < 0.05 vs IFN-γ unstimulated, §*p* < 0.05 vs pSC-UD with IFN-γ by one way ANOVA with Bonferroni’s multiple comparison *post hoc* test (*n* = 3/group).

Parthenogenetic stem cell-derived cells before selection with G418 (undirectedly differentiated cells) and after G418 selection (pSC-derived cardiomyocytes) were treated with mouse recombinant IFN-γ (25 ng/ml) for 48 h to simulate a pro-inflammatory milieu. After treatment with IFN-γ, the cells were dissociated and analyzed by live cell flow cytometry. Unstimulated undirectedly differentiated pSC showed the expression of MHC class I molecules (H2K^d^) on 38 ± 16% of all cells, but no expression of MHC class II molecules. After IFN-γ exposure, MHC class I molecules were expressed on 85 ± 3% and MHC class II molecules on 12 ± 2.3%, of the undirectedly differentiated cells. pSC-derived cardiomyocytes showed very low expression of MHC class I (H-2K^d^) and MHC class II molecules under baseline conditions; IFN-γ treatment resulted in expression of MHC class I and MHC class II molecules on 55 ± 8 and 35 ± 8% of the pSC-derived cardiomyocytes, respectively (Figure 2A). The upregulation of MHC-I on undirectedly differentiated cells and pSC-derived cardiomyocytes was stable for at least 4 days even after withdrawal of IFN-γ (Figure S1 in Supplementary Material).

The PD-1/PD-L1 pathway plays an important role in immune regulation and may contribute to the immune escape of transplanted cells (23). PD-1 was not expressed on undirectedly differentiated pSC and pSC-derived cardiomyocytes even after treatment with IFN-γ. In contrast PD-L1 expression was upregulated by IFN-γ in 33 ± 13% of the undirectedly differentiated pSC and 40 ± 17% of the pSC-derived cardiomyocytes (Figure 2B). No significant upregulation of the co-stimulatory proteins CD40, CD80, and CD86 was observed after IFN-γ-stimulation (Figure 2C). CD1d is a non-classical MHC class I molecule, which is expressed on antigen presenting cells such as dendritic cells, activated monocytes, and B lymphocytes as well as T-cells. It has been reported that CD1d was upregulated on cardiomyocytes during inflammatory conditions like myocarditis and coxsakievirus B3 infections (24). Taking this into consideration, we analyzed the expression of CD1d and found a basal expression of CD1d on 21 ± 17% of all undirectedly differentiated pSC and on 40 ± 9% of all pSC-derived cardiomyocytes. However, CD1d expression was not further induced by treatment with IFN-γ (Figure 2D).

### Functional, Morphological and Immunological Properties of pSC-Derived EHM

**Figure 3:**
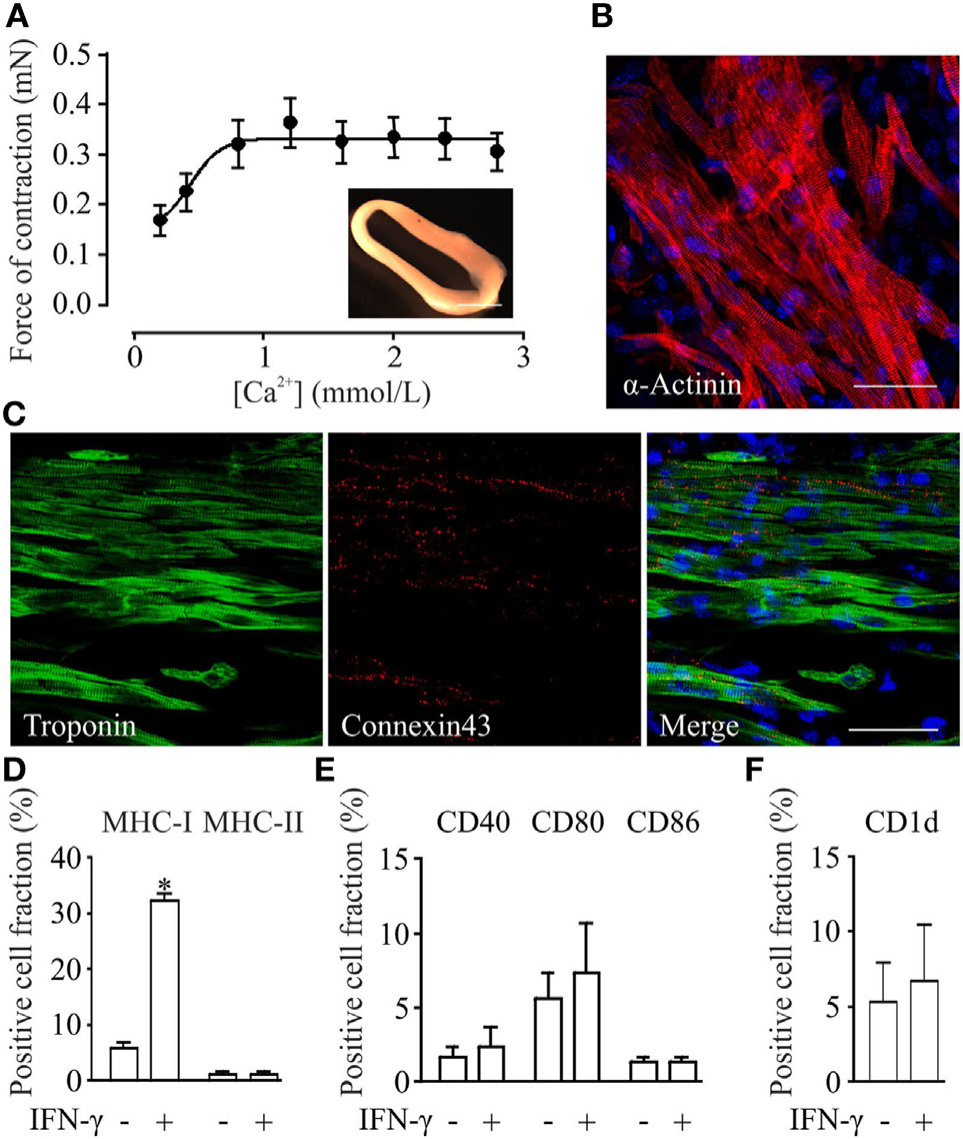
Functional, morphological and immunological properties of parthenogenetic stem cell (pSC)-derived engineered heart muscle (EHM). **(A)** Contractile function of pSC-derived EHM (inset, bar graph: 500 µm) under increasing extracellular [Ca2+] (*n* = 7). **(B,C)** Immunofluorescence stainings of whole mount EHMs. Immunolabeled structures are indicated in the respective panels. DNA (in blue) was labeled with Hoechst. Scale bars: 20 µm. **(D–F)**. FACS-analysis of cells derived from pSC-EHM for major histocompatibility complex (MHC) class I and MHC class II molecules **(D)**, for costimulatory molecules CD40, CD80, CD86 **(E)**, and for CD1d **(F)** with and without IFN-γ stimulation. *p < 0.05 by two-tailed, unpaired Student’s *t*-test (*n* = 3/group).

Engineered heart muscle can be generated from mixtures of pSC-derived cardiomyocytes, inactivated NMRI murine embryonic fibroblasts, and rat tail collagen (20). EHMs showed spontaneous contractions after 4 days in culture (Video S1 in Supplementary Material). Isometric force measurements at culture day 10 demonstrated a positive inotropic response to increasing extracellular calcium concentrations with a contractile force of 0.3 ± 0.04 mN at 2.8 mmol/L extracellular calcium (Figure 3A). Whole mount staining of EHMs for α-actinin showed that pSC-derived cardiomyocytes in EHM are anisotropically arranged (Figure 3B). The EHMs also showed positive staining for the gap junction protein connexin 43 in a diffuse pattern along the cell surfaces, providing morphological evidence for cardiomyocyte coupling within the EHMs (Figure 3C). EHMs were dissociated into single cells and analyzed by flow cytometry. 49 ± 3% (*n* = 7) of the cells could be identified as α-actinin positive cardiomyocytes (Figure S2 in Supplementary Material). Similarly as for pSC-cells obtained directly from spinner flask cultures, EHMs were analyzed under basal and IFN-γ stimulated conditions (25 ng/mL; 48 h prior to dissociation) by flow cytometry. Less than 6% of the EHM-derived cells were positive for MHC class I (H2K^d^), class II (I-A/I-E), CD40, CD80, CD86, and CD1d. Stimulation with IFN-γ increased only the proportion of MHC class I-positive cells (32 ± 1.5%). The percentage of MHC class II-positive did not increase, which contrasted the findings in pSC-derived cardiomyocytes derived directly from cardiac bodies. The observed upregulation of MHC class I molecules was detected specifically on cardiomyocytes since we employed an anti-H2K^d^ antibody which does not detect the alternative H2^q^ haplotypes on the NMRI-murine embryonic fibroblasts (Figure S3 in Supplementary Material). Similar to the observation in spinner flask derived cells, the number of cells positive for CD40, CD80, CD86, or CD1d did not change upon stimulation with IFN-γ (Figures 3D–F).

### Immunostimulatory Capacity of pSC-Derived EHM

**Figure 4:**
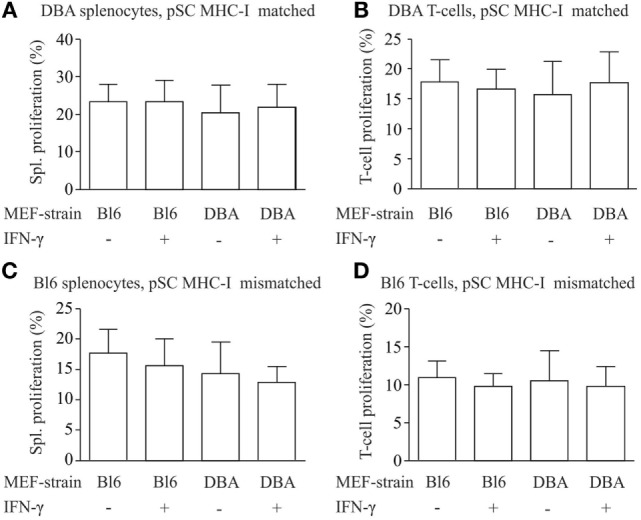
Analysis of immunostimulatory capacity of parthenogenetic stem cell (pSC)-EHM generated from major histocompatibility complex (MHC)-matched or -mismatched pSC-derived cardiomyocytes and murine embryonic fibroblasts. Analysis of proliferation of splenocytes **(A,C)** and T-cells **(B,D)** after co-culture with engineered heart muscles (EHMs) containing MHC-matched **(A,B)** or -mismatched **(C,D)** pSC-derived cardiomyocytes and murine embryonic fibroblasts in presence or absence of IFN-γ. Spl., Splenocytes; Bl6, C57Bl/6J; DBA, DBA/2J (*n* = 3/group); MEF, murine embryonic fibroblasts.

For the analysis of the immune stimulatory properties of pSC-derived EHMs, we employed a co-culture system of EHMs with splenocytes and purified T-cells from DBA/2J (H2K^d/d^) and C57BL/6J-mice (H2^b/b^). Splenocytes and T-cells were stained with eFluor 670, a stable intracellular dye, which upon cell division is distributed equally between the daughter cells. Thus “dilution” of the dye with every cell division can be analyzed by flow cytometry (Figure S4 in Supplementary Material), allowing for a precise assessment of proliferative activity over several cell divisions (25). We generated EHMs from pSC-derived cardiomyocytes derived from pSC (H2K^d/d^) and from inactivated murine embryonic fibroblasts from either DBA/2J mice (H2K^d/d^) or C57BL/6J mice (H2K^b/b^). Inclusion of different murine embryonic fibroblasts in the EHM resulted either in a complete MHC-match or MHC-mismatch for the murine embryonic fibroblasts fraction, allowing analysis of different MHC-match and -mismatch combinations between pSC-derived cardiomyocytes, murine embryonic fibroblasts and responder cells. After 4 days of co-culture, splenocytes were collected and measured for cell proliferation by flow cytometry. DBA/2J and C57BL/6 responder splenocytes co-cultured with DBA/2J-EHMs and C57BL/6J-EHMs did not show significant differences in proliferation, irrespective of IFN-γ treatment or MHC-match situation (Figures 4A–D). Thus, splenocytes and specifically T cells do not proliferate in co-culture with allogeneic EHMs even after treatment of the EHMs with the pro-inflammatory cytokine IFN-γ.

### Retention of pSC-Derived Cardiac Bodies after Implantation under the Kidney Capsules of MHC-Matched or -Mismatched Mice

**Figure 5:**
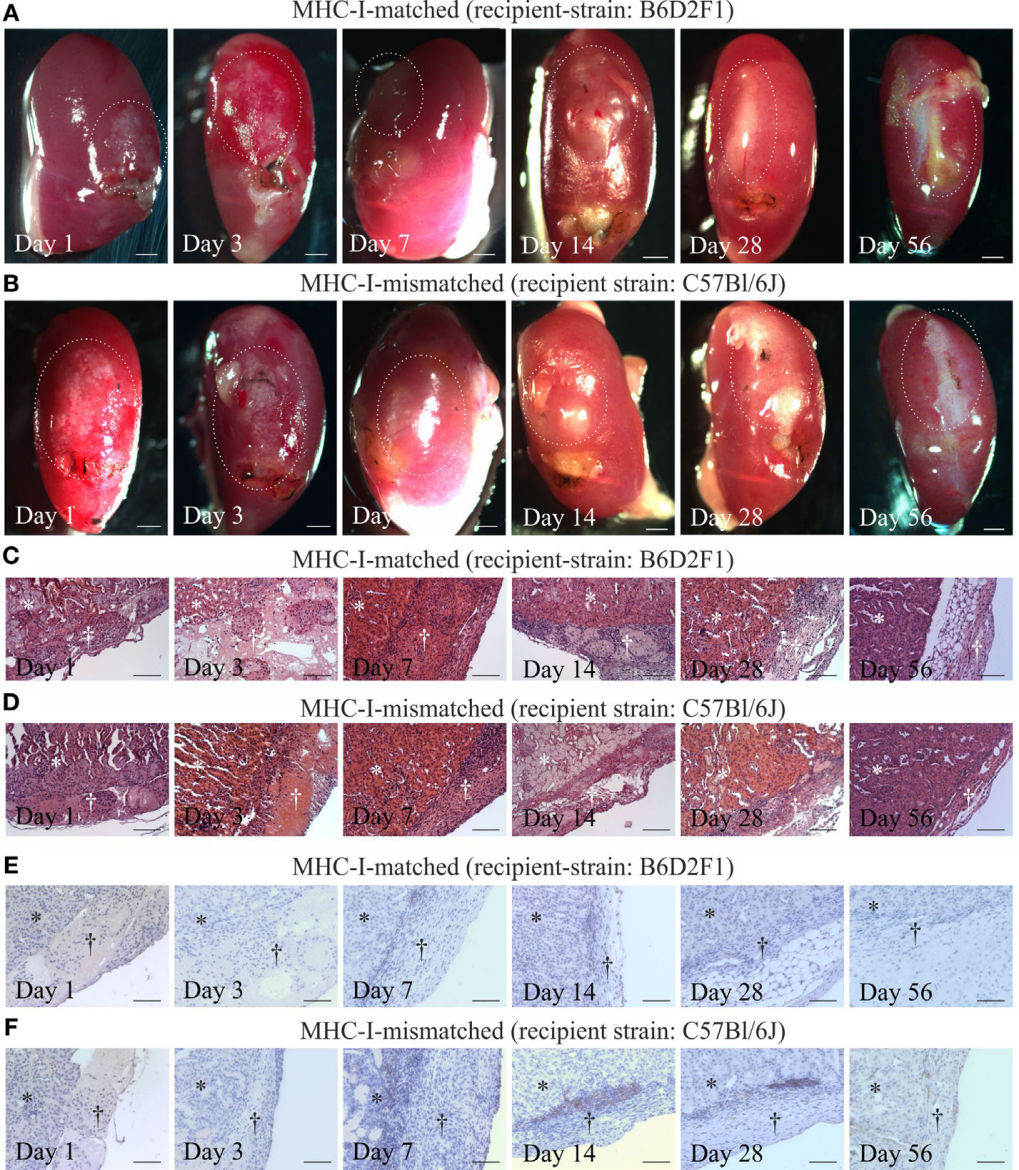
Cardiomyocyte retention and cellular infiltration after implantation of parthenogenetic stem cell-derived cardiac bodies under the kidney capsules of major histocompatibility complex (MHC)-matched or -mismatched mice. Macroscopic images **(A,B)**, hematoxylin & eosin staining **(C,D)**, and immunohistochemistry for CD3 **(E,F)** 1, 3, 7, 14, 28, and 56 days after grafting of cardiac bodies under the kidney capsules in MHC-matched **(A,C,E)** or -mismatched **(B,D,F)** mice. The implantation sites in the macroscopic images are marked by doted lines. In the histological images, the area of the kidney is marked by stars, the area of implantation is marked by crosses (*n* = 2–5/time point). Scale bars: **(A,B)**: 500 µm; **(C–F)**: 100 µm.

Finally, we evaluated the immunogenicity of pSC-derived cardiomyocytes in mice with an MHC-matched (B6D2F1, H2^b/d^) and MHC-mismatched (C57BL/6J, H2^b/b^) background. B6D2F1 mice were chosen to simulate the anticipated clinical scenario of a transplantation of MHC class I homozygous pSC-derivatives (H2^d/d^) into recipients with a heterozygote MHC genotype (e.g., H2^d/b^) (20); in this transplant scenario, the graft shows a full match to the recipient but lacks one of the recipients MHC class I haplotypes. pSC-derived cardiac bodies were implanted underneath the kidney capsule. Kidneys were harvested 1, 3, 7, 14, 28, and 56 days after implantation and monitored for graft survival and cellular infiltration. Spontaneous contraction could be observed macroscopically until day 3 and in one animal until day 28 after implantation under MHC-matched conditions and until day 7 under MHC-mismatched conditions (Figures 5A,B; Videos S2 and S3 in Supplementary Material). The implants could be identified in hematoxylin & eosin stainings (Figures 5C,D). Three of five mice at day 28 and two of three mice at day 56 developed large teratoma in case of a MHC-matched transplant (Figure S5 in Supplementary Material; Table 1). No teratoma formation was observed in the MHC-mismatched transplantations. Furthermore, we analyzed the infiltration of the implants with CD3-positive T-cells by immunohistochemistry. In the mismatched conditions in some animals we observed small clusters of CD3-positive cells at the border zone between implant and kidney. However, only very few CD3-positive cells were found inside the implants, even in the MHC-mismatched conditions (Figures 5E,F; Figures S6 and S7 in Supplementary Material).

**Table 1:** Parthenogenetic stem cell-derived cardiac bodies after implantation into kidney capsules of major histocompatibility complex (MHC)-matched and -mismatched mice.

	Days after implantation					
	1	3	7	14	28	56
MHC-matched	2/2/0	2/2/0	3/0/0	3/1/0	5/1/3	3/0/2
MHC-mismatched	2/2/0	3/3/0	2/2/0	3/0/0	5/0/0	3/0/0

*Overview of n-number of implants/implants showing spontaneous beating at the time-point of explantation/implants having formed teratoma at the time-point of explantation*.

## Discussion

### MHC-Expression and Immunogenicity of Native- and Stem Cell-Derived Cardiomyocytes

We analyzed the immunological properties of pSC-derived cardiomyocytes and pSC-EHM. The expression of allogeneic MHC-molecules by antigen presenting cells is one of the main mechanisms of allograft rejection (10). Here, we demonstrate that pSC-derived, unselected cells showed a high expression of MHC class I molecules. This is similar to derivatives of embryonic stem cells in which the increased expression of MHC class I molecules after differentiation is accompanied by an increased immunogenicity (26, 27). pSC-derived cardiomyocytes, in contrast, expressed MHC class I and several other immune-modulatory molecules only at a very low level. Even after further morphological maturation in a complex three-dimensional EHM-culture format in co-culture with murine embryonic fibroblasts, the expression levels of these molecules did not increase. This phenotype might contribute to our findings, that EHM did not induce splenocyte or T-cell proliferation *in vitro* despite a complete MHC-mismatch. In line with our *in vitro* observation, Rosa et al. showed that native myocardium does not express MHC class I antigens, but after transplantation these antigens can be induced (28). It was demonstrated that fetal and adult myocytes express very low levels of MHC class I antigens and do not have detectable levels of class II antigens (29). On the other hand, endothelial cells lining the microvasculature express both MHC class I and class II antigens (30).

Heart transplants, when compared to other organs such as skin or pancreas, are associated with a relatively low level of immune rejection (31). This may be attributed to a low level of MHC class I molecule expression, but does finally not result in heart transplant retention into MHC disparate recipients (32). This rejection is the consequence of an acute immune response typically directed against the endothelial lining of the transplant vasculature. Moreover, tissue resident professional antigen presenting cells such as macrophages, dendritic cells and B cells that are transplanted with the hearts can initiate a direct allogeneic immune response. Only few studies (33) have investigated the immunological properties of isolated cardiomyocytes and to our knowledge no prior study has analyzed the immunological properties of isolated stem cell-derived cardiomyocytes, so far.

### Potential Mechanisms of Reduced Immunogenicity in pSC-Derived Cardiomyocytes

After stimulation with IFN-γ, pSC-derived cardiomyocytes upregulated MHC class I and class II molecules. In EHMs, IFN-γ did not upregulate MHC class II molecules. This could be interpreted as a sign of “immunological maturation” accompanying the structural maturation during the three-dimensional EHM-culture (20) since in adult cardiomyocytes, as in most non-professional antigen presenting cells, MHC class II molecules are not expressed even under pro-inflammatory stimuli (34). In contrast to MHC class II molecules, the expression of MHC class I antigens was highly upregulated also in EHM after IFN-γ stimulation. In view of a potential clinical application of EHM and its implantation into the pro-inflammatory milieu of an infarcted myocardium, this has to be considered as a potential challenge. Nevertheless, even IFN-γ-treated EHM did not induce a splenocyte or T-cell proliferation *in vitro*. There are several cellular mechanisms that can lead to an escape from immune rejection despite MHC-mismatch (10). For example, the absence of co-stimulatory molecules on the transplanted cells will render T-cells anergic, i.e., non-responsive to stimulation by antigens, that are capable of direct allorecognition (35). In this study, we could detect only a very low expression of co-stimulatory molecules (CD40, CD80 and CD86) on pSC-derived cardiomyocytes and EHMs even after stimulation by IFN-γ. Another important mechanism regulating the peripheral tolerance and autoimmunity is the expression of PD-L1 (36, 37). Binding of PD-1 on T cells to PD-L1 results in the activation of suppressive functions such as T-cell anergy or induction of programmed cell death as well as stimulation of regulatory T-cells. It has been shown that expression of PD-1 and PD-L1 was significantly increased in ischemic-reperfused hearts resulting in an inhibition of T-lymphocyte proliferation, but also in an increase in cardiomyocyte death *via* an autocrine mode of action (38). Interestingly, we found an upregulation of PD-L1 on pSC-derived cardiomyocytes after IFN-γ stimulation that could lead to T-lymphocyte inhibition despite an upregulation of MHC class I molecules.

Purified cardiomyocytes will not form a three-dimensional syncytium without non-cardiomyocytes. In a clinical application autologous non-cardiomyocytes could be acquired, for example by expanding fibroblasts from skin-biopsies. To simulate a clinical scenario, we generated EHMs from pSC-derived cardiomyocytes and murine embryonic fibroblasts with disparate MHC-haplotypes. Unexpectedly, MHC-mismatch of the murine embryonic fibroblasts did not induce a splenocyte or T-cell proliferation *in vitro*. It is unclear if this is a unique feature of murine embryonic fibroblasts or if EHMs can provide an immunoprotective milieu.

### Survival and Teratogenicity of pSC-Derived Cardiomyocytes *In Vivo*

Since our *in vitro* results indicated a reduced immunogenicity of pSC-derived cardiomyocytes, we aimed at testing the immune rejection of pSC-CM in non-immunosuppressed MHC-matched and mismatched mice. Beating of implanted cardiac bodies could be observed until day 7 even under MHC-mismatched conditions. Microscopically, the implants could be identified at least until day 56. We found an infiltration with few CD3-positive T cells-clusters mainly at the border zone between implant and kidney and only few cells infiltrating the implant itself in the MHC-mismatched setting. In contrast, in studies using MHC-mismatched whole heart transplants a complete, CD4^+^ and CD8^+^ T-cell mediated rejection of the transplants occurs within 16 days after transplantation (32).

After implantation of purified pSC-derived cardiomyocytes into the kidney capsule, teratoma formation was observed after 28 days only in MHC-matched recipients. This indicates on the one hand the presence of undifferentiated pSC even after cardiomyocyte selection and confirms on the other hand allograft survival (20). A higher degree of cardiomyocyte purity (>90%) and prolonged culture to enhance differentiation of remaining stem cells will be particularly important under MHC-matched conditions to prevent tumor growth. In fact, Hentze et al. could demonstrate that as few as 245 pluripotent stem cells can lead to teratoma formation (39). Conversely, human pSC-derived neural stem cells did not induce teratomas in immune deficient athymic nude rats (40). This is in line with own data on the implantation of human embryonic stem cell derived EHM in nude rats (41).

## Conclusion

In conclusion, pSC-derived cardiomyocytes represent an interesting cell source for allogeneic heart repair applications. Despite MHC-matching, mild immune responses are expected. Whether and what type of immune control will be needed remains to be investigated. Data from the ongoing first clinical trials on embryonic stem cell-, iPSC-, and pSC-derived cell implantation will provide invaluable information on the potential limitation of immune responses and on how to counter them for effective repair.

## Ethics Statement

All animal experiments were performed according to institutional and governmental guidelines and approved by the Niedersächsisches Landesamt für Verbraucherschutz und Lebensmittelsicherheit (LAVES; 33.9.42502-04-11/0606, -13/1300, -15/1841).

## Author Contributions

MD, RD, and W-HZ designed the study; MD, SG, VM, and RD acquired data; MD, SG, VM, RD, and W-HZ analyzed and interpreted data; MD drafted the manuscript; RD and W-HZ revised the manuscript; all authors approved the final version of the manuscript.

## Conflict of Interest Statement

The authors declare that the research was conducted in the absence of any commercial or financial relationships that could be construed as a potential conflict of interest.

## Supplementary Material

Video S1 Spontaneously beating parthenogenetic stem cell-derived engineered heart muscle on culture day 12.

Video S2 Parthenogenetic stem cell-derived cardiac bodies 28 days after implantation under the kidney capsule of a major histocompatibility complex-matched B6D2F1 mouse showing spontaneous beating activity.

Video S3 Parthenogenetic stem cell-derived cardiac bodies 7 days after implantation under the kidney capsule of a major histocompatibility complex-mismatched C57BL/6J mouse showing spontaneous beating activity.

## Abbreviations

B6D2F1, C57Bl/6J × DBA/2J F1-hybrid; EHM, engineered heart muscle; FACS, fluorescence-activated cell sorting; IFN-γ, interferon gamma; iPSCs, induced pluripotent stem cells; MHC, major histocompatibility complex; PBS, phosphate buffered saline; pSCs, parthenogenetic stem cells.
