# Supplementary material for: Immunological Properties of Murine Parthenogenetic Stem Cell-Derived Cardiomyocytes and Engineered Heart Muscle
Source: Front Immunol. 2017 Aug 14;8:955. doi: 10.3389/fimmu.2017.00955 (PMC5557729; doi:10.3389/fimmu.2017.00955)
Supplement: Supplementary file 1 [file data_sheet_1.pdf]

Supplementary Material

Immunological properties of murine parthenogenetic stem cell derived cardiomyocytes and engineered cardiac muscle

Michael Didié, Satish Galla, Vijayakumar Muppala, Ralf Dressel, Wolfram-Hubertus Zimmermann

- Supplementary Videos 1 to 3
- Supplementary Figures 1 to 7

Supplementary Video 1: Spontaneously beating pSC-derived EHM on culture day 12.

Supplementary Video 2: pSC-derived cardiac bodies 28 days after implantation under the kidney capsule of a MHC-matched B6D2F1 mouse showing spontaneous beating activity.

Supplementary Video 3: pSC-derived cardiac bodies 7 days after implantation under the kidney capsule of a MHC-mismatched C57BL/6J mouse showing spontaneous beating activity.

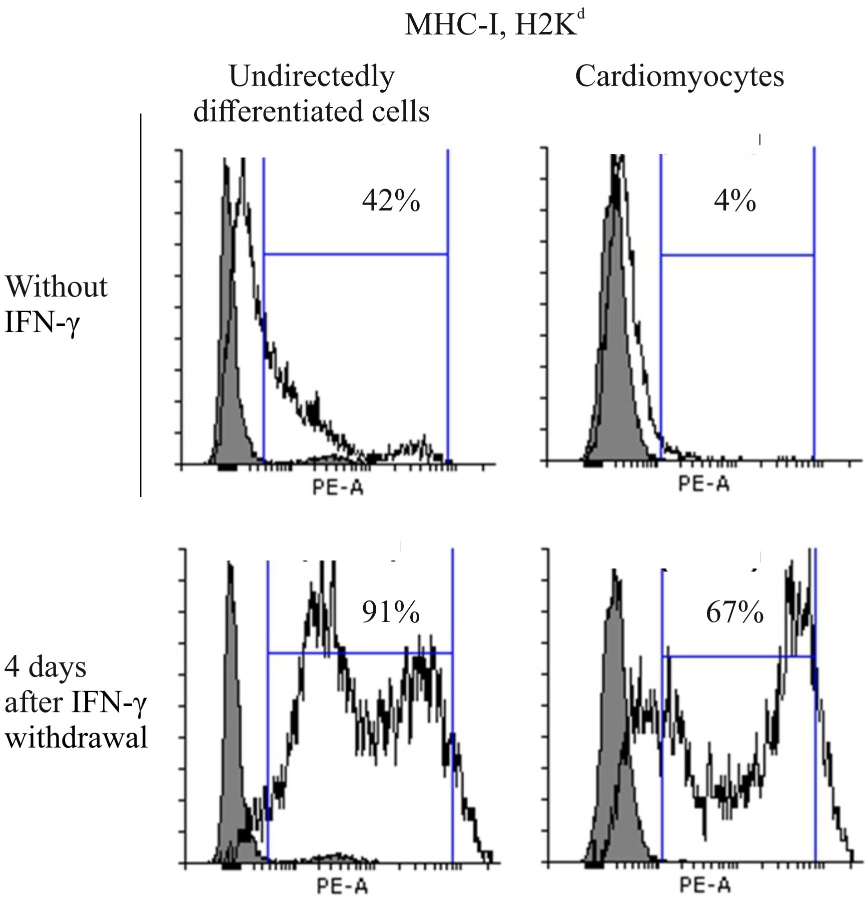

Supplementary Figure 1: Sustained MHC-I expression after transient IFN-γ stimulation. pSC-derived undirectedly differentiated cells and pSC-derived cardiomyocytes were stimulated with IFN-γ for 48 h. MHC-I upregulation could be detected even 4 days after IFN-γ withdrawal by FACS-analysis. Grey histograms represent isotype controls.

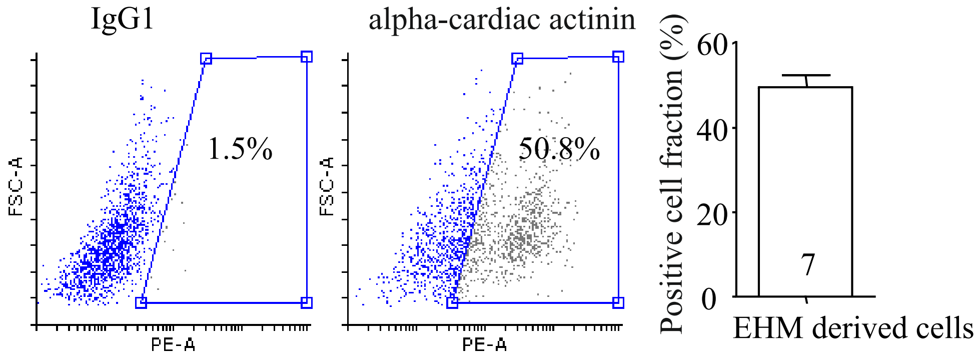

Supplementary Figure 2: Cardiomyocyte content in pSC-derived EHMs. Representative scatter blots of FACS-analysis after dissociation of EHMs into single cells and staining with IgG1-control antibodies (left panel) and antibodies against alpha-cardiac actinin (middle panel). The bar graph shows the average cell fraction of 7 analyzed EHMs (right panel).

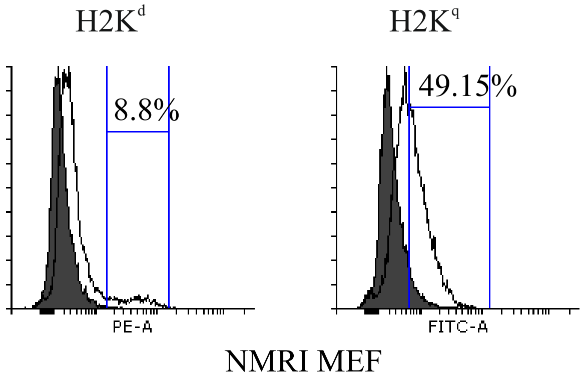

Supplementary Figure 3: Staining of MEFs to demonstrate cardiomyocyte specific staining of MHC class I molecules in EHMs generated from pSC-derived cardiomyocytes (H2^d) and MEFs derived from NMRI-mice (H2^q). FACS analysis of NMRI-MEFs labeled with an H2K^d-antibody (left panel) and an H2K^q-antibody (right panel). Black histograms represent IgG-controls.

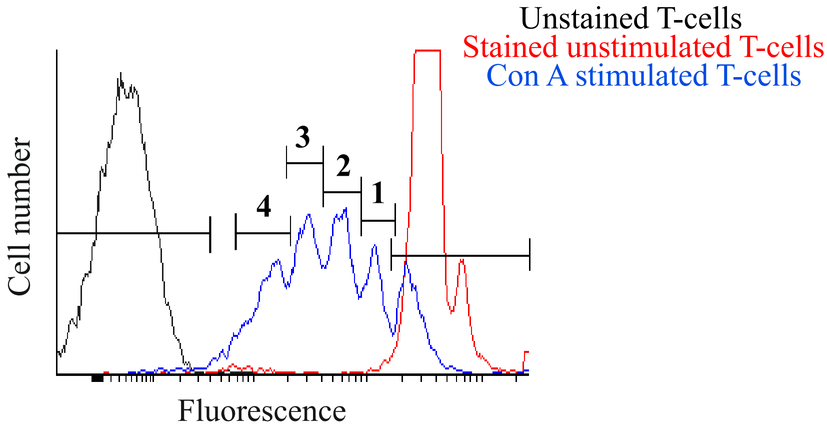

Supplementary Figure 4: Proliferation assay with eFluor670 labeled T-lymphocytes. Treatment of purified eFluor670 (APC-A) T-lymphocytes isolated from Balb/c spleen with the unspecific T-cell stimulator concanavalin A for 4 days resulted in up to 4 consecutive cell divisions with a reduction of fluorescence intensity by 50% with each cell division (see peaks in histogram).

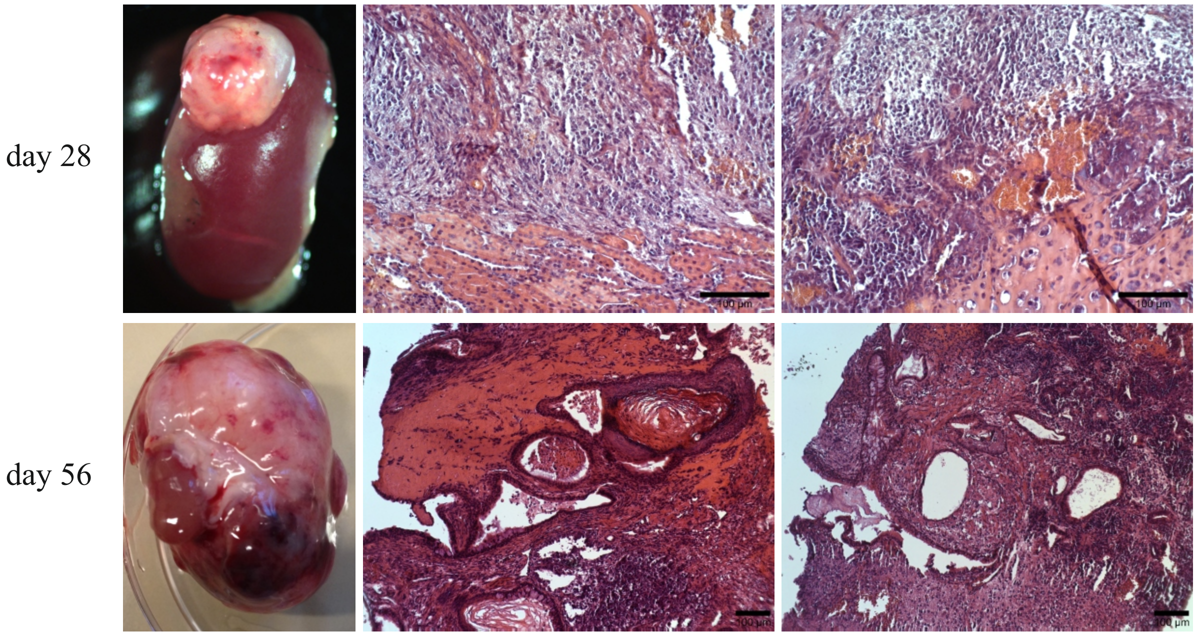

Supplementary Figure 5: Teratoma-induction after implantation of pSC-derived cardiac bodies under the kidney capsule of MHC-matched mice. Macroscopic views (left panels) and hematoxylin & eosin staining (middle and right panels) of representative teratomas 28 and 56 days after implantation.

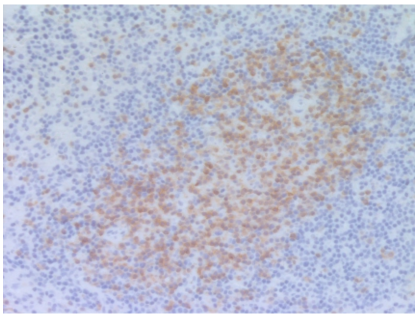

Supplementary Figure 6: Control staining of CD3-posive cells. Staining of CD3-expressing cells in the spleen.

A

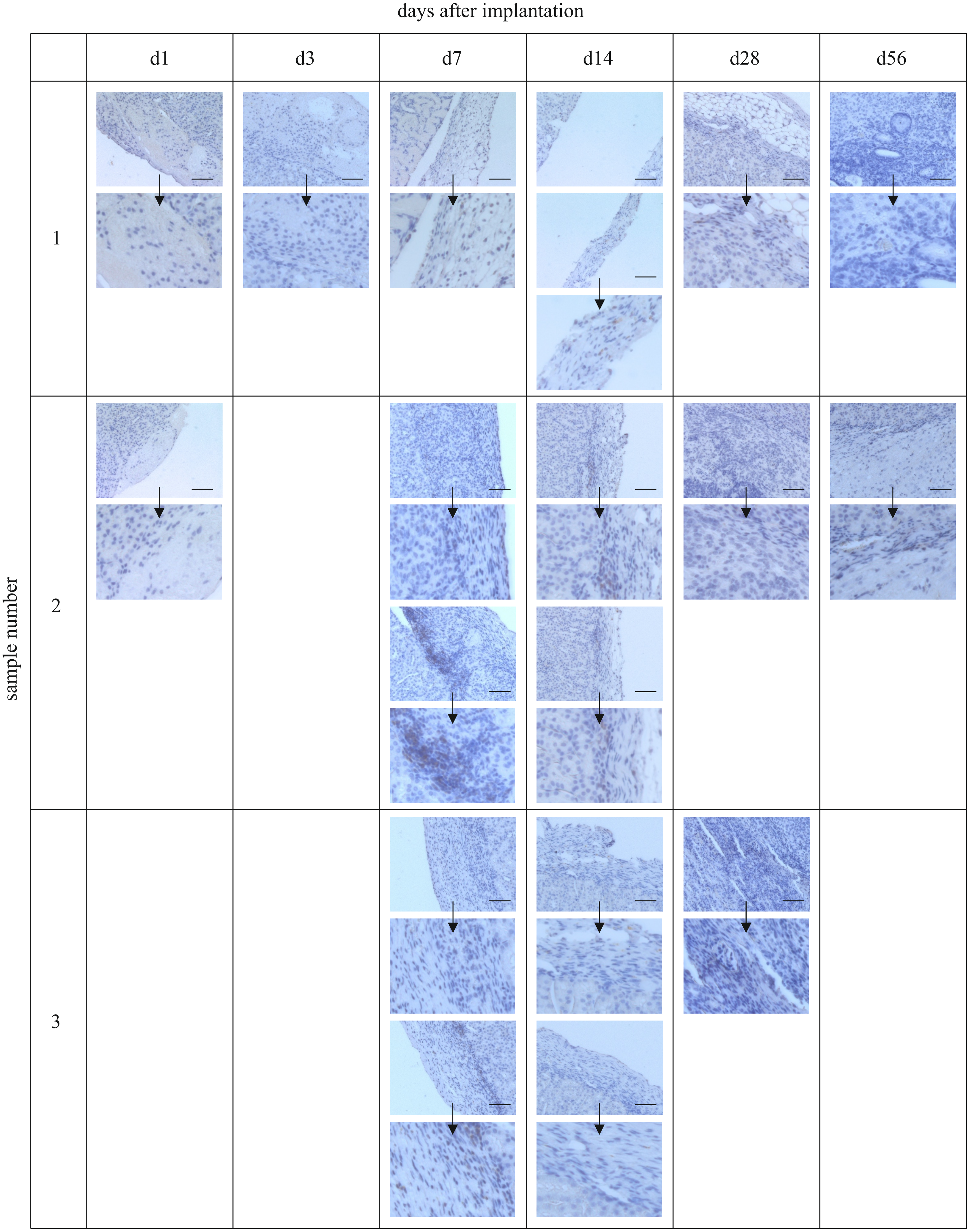
days after implantation
d1
d3
d7
d14
d28
d56
sample number
1
2
3

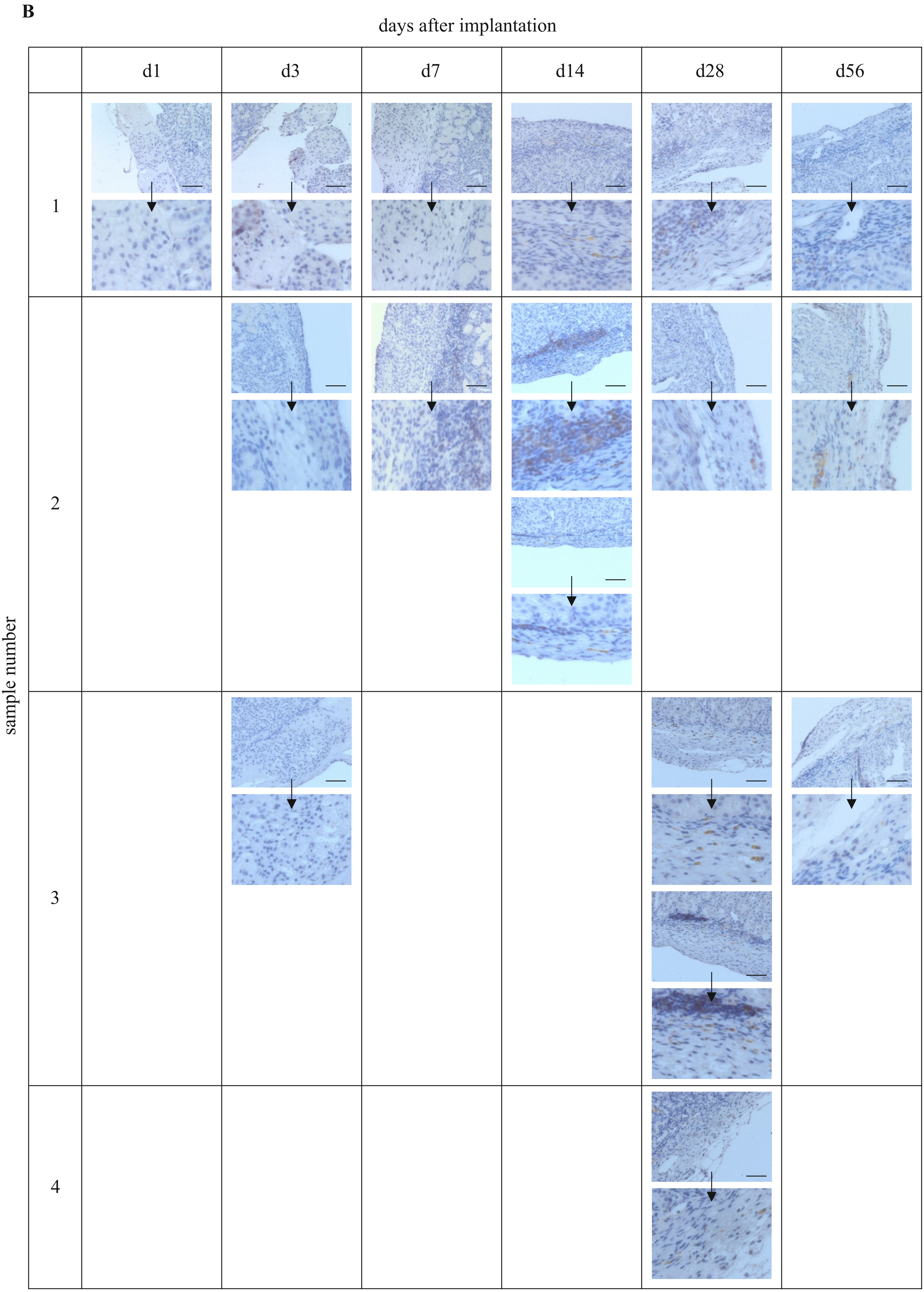
B
days after implantation
d1
d3
d7
d14
d28
d56
sample number
1
2
3
4

Supplementary Figure 7: Immunohystochemistry for CD3 of pSC-derived cardiac bodies after implantation under the kidney capsule of MHC -matched or mismatched mice. Images of all samples analyzed at the indicated days after implantation are presented. In some samples multiple images are shown. Magnifications of areas at the border zones between implants and kidney are presented and indicated by arrows. (**A**) Cardiac bodies implanted under the kidney capsules of MHC-matched (B6D2F1-hybrids) mice. (**B**) Cardiac bodies implanted under the kidney capsules of MHC-mismatched (C57BL/6J) mice.
